# Factors associated with hospitalization for aortic stenosis in Portugal from 2015 to 2017

**DOI:** 10.1093/eurpub/ckac131.021

**Published:** 2022-10-25

**Authors:** FG Avelar, J Alves

**Affiliations:** 1 Public Health Research Centre, National School of Public Health, Lisbon, Portugal; 2 Comprehensive Health Research Centre, Lisbon, Portugal

## Abstract

**Background:**

Severe aortic stenosis prevalence has been growing worldwide and constitutes a public health challenge. The gold-standard treatment is Surgical Aortic Valve Replacement (SAVR) however Transcatheter Aortic Valve Implantation (TAVI) has been increasing, especially in high-risk surgical patients. This study aims identifying the factors associated to the implementation of TAVI to minimize possible disparities in access to health services.

**Methods:**

This study used data on inpatient discharges from the Portuguese NHS, from 2015 to 2017. SAVR and TAVI, were classified according to the International Classification of Diseases (ICD). Chi-square test and independent T-tests with 1% significance level in the SPSS® were performed to identify the factors associated with both interventions.

**Results:**

A total of 8398 hospitalizations were analysed, 88.5% SAVR and 11.5% TAVI. The mean (SD) age for SAVR was 70 (±11) years old and 81 (±7) years old for TAVI (p < 0.001), 56.9% were male among SAVR and 44.6% among TAVI (p < 0.001). Year (p < 0.001), type of admission (p < 0.001), geographic location (p < 0.001), severity (p < 0.001) and Charlson Comorbidity Index (CCI) (p < 0.001) were associated with the type of intervention.

**Conclusions:**

TAVI was performed in more severe patients and there was an increase in TAVI over the years, which is consistent with the growing use of the technology among other patients, e.g., the high-risk surgical patients. We also found a geographic pattern in the use of SAVR and TAVI. This might reveal the existence of geographic disparities regarding availability and access to health services.

**Key messages:**

• In Portugal, there is an increase in the performance of TAVI, with geographical concentration that reflects on access.

• TAVI is more often performed in more severe patients as an alternative to SAVR with similar discharge outcomes.

